# Assessing Residency Program Approaches to the Transgender Health CREOG Objective

**DOI:** 10.1089/trgh.2015.0011

**Published:** 2016-03-01

**Authors:** Frances W. Grimstad, Catherine L. Satterwhite, Carrie L. Wieneke

**Affiliations:** ^1^ Department of Obstetrics and Gynecology, University of Kansas Medical Center, Kansas City, Kansas.; ^2^ Department of Preventative Medicine and Public Health, University of Kansas Medical Center, Kansas City, Kansas.

**Keywords:** health education/training programs, transgender

## Abstract

**Purpose:** The transgender population is a small yet distinctive portion of the gynecology patient population, requiring both primary care and specialty services. Recognizing the need for increased education, the Council on Resident Education in Obstetrics and Gynecology (CREOG) developed objectives specific to the care of transgender patients. This study is to assess residency program directors' knowledge about the transgender health CREOG objectives, describe how objectives are being implemented in training programs, and identify what types of educational materials would be useful if available.

**Methods:** In May 2014, an 11-item anonymous survey was sent through e-mail to all eligible program directors of accredited obstetrics and gynecology residency programs. The short questionnaire contained questions about program demographics, approach to training residents with regard to the CREOG objectives, and opinions on tools they would like to use to train their residents on the transgender CREOG objectives.

**Results:** Just under half (47%) of the 86 geographically diverse respondents were from hospital-based programs. The majority reported that the transgender health objectives were important (82%); however, only 70% were familiar with the objectives themselves. Most respondents (96%) felt that providing an educational activity in their training program would be beneficial for their residents' education.

**Conclusions:** Most program directors support the CREOG transgender health objectives and are in favor of implementing educational tools to meet the objectives, suggesting that development of new tools to meet this need would be useful. Future endeavors will be made toward build a training module to facilitate obstetrics and gynecology (Ob-Gyn) programs meeting the CREOG objectives.

## Introduction

The transgender population is a small yet distinct portion of the gynecology patient population, requiring primary care as well as specialty gynecologic services. An estimated 0.5–2% of the general population is transgender.^1^ The healthcare needs of the transgender population are substantial. In a 1997 survey done by the San Francisco Department of Public Health, 35% of male-to-female transgender persons tested positive for HIV, and roughly 85% of transgender patients reported verbal abuse because of their gender identity or presentation.^2^ The National Transgender Discrimination Survey, a 2008 survey of 6436 transgender persons, found that at least 25% of respondents reported being harassed or disrespected in a doctor's office or hospital, and 19% were refused medical care.^3^ More than one-quarter (28%) of transgender individuals postponed seeking healthcare, even while ill, because of discrimination. To address these issues, half of respondents acknowledged the need to teach their healthcare providers about transgender care.

Many organizations are recognizing this need and vocalizing the importance of increased education to build the skills appropriate for addressing the unique health concerns of transgender individuals. In particular, the World Professional Association for Transgender Health (WPATH) has been a vocal proponent of increased access to healthcare for transgender persons as well as improved knowledge of transgender health issues among practicing physicians.^4^ In 2011, The American College of Obstetricians and Gynecologists also echoed the need for education on transgender identity and healthcare in a committee opinion.^5^

**FIG. 1.**
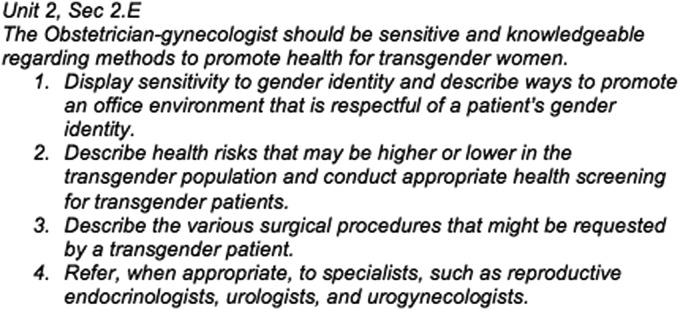
The Transgender Health CREOG objective, broken down into its four key parts.^8^ CREOG, Council on Resident Education in Obstetrics and Gynecology.

Obstetrics and Gynecology (Ob-Gyn) residency training provides a timely opportunity to address transgender health with the next generation of gynecologists, allowing them to feel competent caring for these patients when they begin practicing as independent physicians. The Council on Resident Education in Obstetrics and Gynecology (CREOG) objectives (Fig. 1) for residency education that focus on transgender health include knowledge of transgender health issues and an understanding of when and how to refer appropriately.

To better understand how these objectives are used in residency programs, residency program directors were surveyed to assess their knowledge of the CREOG objectives specific to transgender health, how they implement related training in their programs, and what forms of training materials they would use if they were made available. Our hypothesis was program directors were unaware of the Transgender Health CREOG objectives and were thus not implementing training tools in their programs.

## Methods

This study was reviewed and approved by the University of Kansas Medical Center Institutional Review Board; appropriate informed consent was obtained from all subjects.

The study population included all Ob-Gyn residency program directors practicing within the contiguous United States with programs recognized by the American College of Obstetricians and Gynecologists. All program directors were invited to participate in the anonymous survey through e-mail. The e-mail addresses were identified from the circulating listserv of Ob-Gyn program directors as of May 2014.

**FIG. 2.**
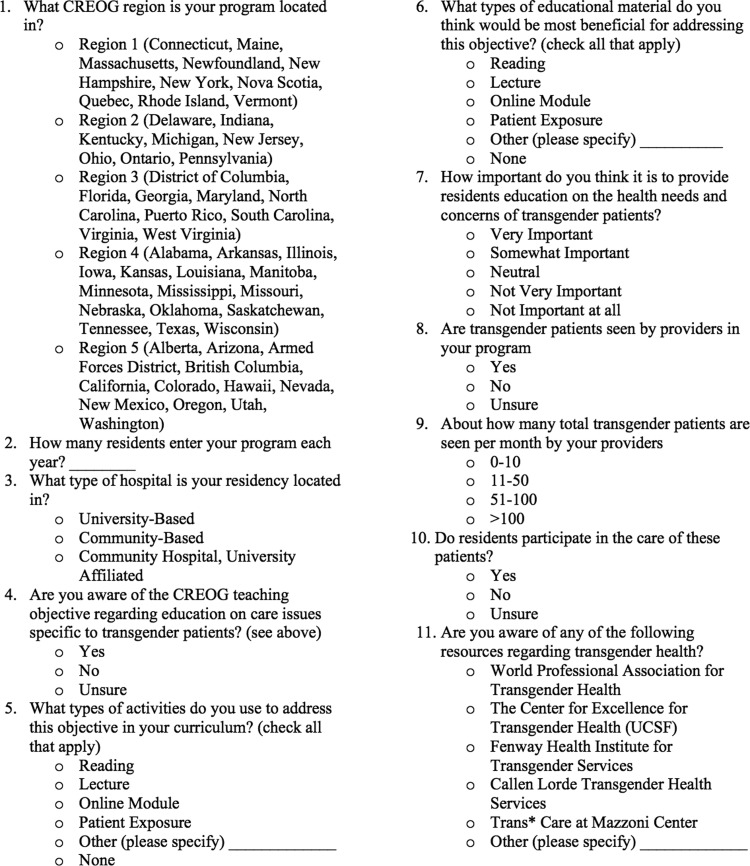
The survey that was provided to the program directors.

As there is no validated survey currently in circulation to assess residency program directors awareness of CREOG objectives, a team consisting of a program director, resident physician with experience in transgender health and advocacy, and an epidemiologist were used to construct the survey (Fig. 2). While the primary goal was to understand the awareness level of the objective, other questions were chosen to further elucidate how programs were meeting these objectives and what their ideal setting would be to address the objectives. Finally, basic demographic questions were requested so that correlations could be made between program characteristics and approaches to the objectives. For most questions with “yes/no” answers (Fig. 2, question 4, 8, and 10), an “unsure” option was provided to acknowledge those program directors who were not specifically aware of the transgender component of their residency. Questions regarding educational tools (Fig. 2, question 5 and 6) were given the most common responses as per academic experience, with a line allowing for alternative methods to be explained. Solicitation for how important the objectives were was asked on a Likert scale (Fig. 2, question 7). The question (Fig. 2, question 9) regarding the number of transgender patients seen in the residency program was grouped to try and capture the best estimation by the program director. The survey was circulated among a small number of academic faculty who were not familiar with the study to solicit feedback on readability and ease of response.

There were 219 Ob-Gyn residency program directors eligible to participate at the time of the study. The survey was sent out as a link in the e-mail body. Any e-mail addresses from the initial invitation that were returned as a nonworking address were placed into a separate category, and an attempt was made to determine which programs these addresses were associated with. If that determination could be made, then an Internet search was used to ascertain if a more current e-mail could be found. A general follow-up e-mail that included the updated e-mails was sent 1 week later to all program directors to encourage participation. Two weeks after the follow-up e-mail, a final e-mail was sent to a listserv of program coordinators with the request to pass along the e-mail to their current program director to encourage further responses.

A statement of release of data was issued at the beginning of the survey; the participant assented to the use of data and participation in the study by completing the survey. The survey was voluntary, and there was no compensation. The short survey was administered through Survey Monkey and consisted of 11 items regarding program demographics, the approach to training residents with regard to the CREOG transgender objectives, and opinions on tools program directors would like to use to train their residents in the CREOG objectives.

Data from the survey were analyzed using SAS (Version 9.4, Cary, NC). To test for differences between proportions, chi-square tests or Fisher's exact tests were used.

## Results

Of the 219 eligible program directors, 86 responded to the survey (39.3%). The median number of residents in these programs was 5 (mean: 6), with a median range of 4–5.5 residents across CREOG regions. Overall, more than three-quarters of programs were either university based or located in a community hospital setting with a university affiliation (Table 1).

**Table 1.** **Survey Responses by CREOG Region and Overall**

CREOG region	1	2	3	4	5	Overall
Number of responses	16	17	16	23	14	86
Average no. of residents	6	7	7	5	5	6
Location of program (%)						
University based	68.8	23.5	50.0	52.2	35.7	46.5
Community based	6.3	29.4	25.0	13.0	42.9	22.1
Community-based university affiliated	25.0	47.1	25.0	34.9	21.4	31.4
Aware of objectives (%)						
Yes	75.0	52.9	75.0	69.6	92.9	72.1
No	6.3	23.5	12.5	26.1	7.1	16.3
Unsure	18.8	23.5	12.5	4.4	0.0	11.6
Importance of objectives (%)						
Very important	43.8	17.7	25.0	34.8	28.6	30.2
Somewhat important	37.5	52.9	50.0	47.8	71.4	51.2
Neutral	12.5	17.7	0.0	8.7	0.0	8.1
Not very important	6.3	5.9	25.0	4.4	0.0	8.1
Not important	0.0	5.9	0.0	4.4	0.0	2.3
Transgender patients seen in clinic (%)						
Yes	62.5	25.0	62.5	39.1	42.9	45.9
No	12.5	31.3	18.8	39.1	35.7	28.2
Unsure	25.0	43.8	18.8	21.7	21.4	25.9

CREOG regions:

1. Connecticut, Maine, Massachusetts, Newfoundland, New Hampshire, New York, Nova Scotia, Quebec, Rhode Island, Vermont.

2. Delaware, Indiana, Kentucky, Michigan, New Jersey, Ohio, Ontario, Pennsylvania.

3. District of Columbia, Florida, Georgia, Maryland, North Carolina, Puerto Rico, South Carolina, Virginia, West Virginia.

4. Alabama, Arkansas, Illinois, Iowa, Kansas, Louisiana, Manitoba, Minnesota, Mississippi, Missouri, Nebraska, Oklahoma, Tennessee, Texas, Wisconsin.

5. Alberta, Arizona, Armed Forces District, British Columbia, California, Colorado, Hawaii, Nevada, New Mexico, Oregon, Utah, Washington.

To test for differences between proportions, chi-square tests or Fisher's exact tests were used. Continuous variables were evaluated using a two-sample *t*-test.

CREOG, Council on Resident Education in Obstetrics and Gynecology.

**FIG. 3.**
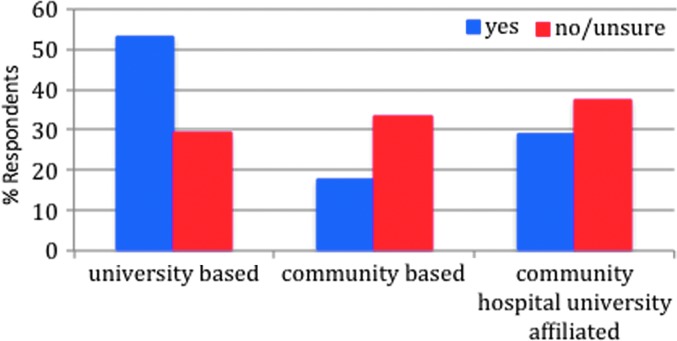
The awareness of objectives based on hospital type (by percentage). The majority of program directors who were aware of the objectives came from university-based programs, while those who were unaware of the objectives were primarily from community-based and university-affiliated hospitals.

**FIG. 4.**
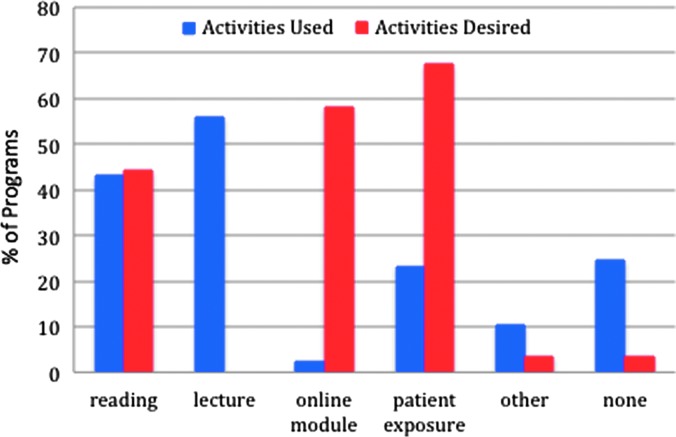
The percentage of Ob-Gyn programs that use, versus desire, each activity. The majority of programs reported using reading and lectures to fulfill the CREOG objective. Program directors, however, would prefer to have online modules or patient exposure. Ob-Gyn, Obstetrics and Gynecology.

The majority of program directors reported awareness of the CREOG transgender objectives (72.1%). While there was no statistically significant difference by region, reported awareness appeared to be lowest in Region 2 (52.9%), compared to Region 5, where 92.9% of program directors reported awareness of the objectives (Table 1). Although not statistically different, data suggested that program directors of university-based programs were more frequently aware of the objectives (82.5%) than program directors of university-affiliated programs (66.7%) and program directors of community-based programs (57.9%) (*p*=0.21) (Fig. 3). Among respondents who were aware of the objectives, the most commonly used supportive educational activities were lectures (62.9%) and reading materials (51.6%) (Fig. 4). However, 14.5% of respondents who reported awareness of the CREOG objectives reported that they used no modality for educating their residents (lecture, reading, online module, patient exposure, or other).

Direct patient exposure was felt to be the most desirable form of education (67.4%), however, this was not widely available. Only 45.9% of respondents stated that transgender patients were seen in their clinics, while only 8.1% stated that their clinics saw more than 10 transgender patients per year. Of those who saw transgender patients, only 36.9% reported that residents participated in transgender patient care. Overall, only 23.3% stated they utilized direct patient exposure as a learning tool. After direct patient exposure, the next type of material reported to be beneficial was an online module (58.1%).

Regardless of the method of exposure, there was consensus that the CREOG objectives were important. More than 80% of respondents ranked the objectives as important, with 37.5% of those stating it was very important. Only 2.3% of program directors indicated that the objectives were not important at all. While there are some resources available that can be used to educate residents about transgender health, few program directors reported being aware of them. Of the 86 programs, only 13 were familiar with the World Professional Association with Transgender Health (15.1%), and only 26 knew about the University of California at San Francisco Center for Excellence for Transgender Health (30.2%).

## Discussion

The CREOG objectives form the basis of a well-rounded Ob-Gyn residency graduate. Despite the recognized need for training on the healthcare needs of transgender patients, only three-quarters of participating program directors reported being aware of the CREOG objectives pertaining to the care of transgender individuals. In addition, despite 81.4% of respondents indicating that the objectives were important, roughly a quarter of respondents reported that they did not have any modality for education.

While there is an opportunity to increase awareness of the CREOG transgender objectives, there are few educational tools to adequately equip program directors to provide this training. While the ideal is an increase in patient interaction, there is little direct control that programs can have over increasing their transgender patient population. In addition, residents in a program with a recognized transgender patient population may still have limited access to that population. Only 36.9% of program directors reported that their residents had access to direct patient observation. An alternate method should be sought to provide education. Online models and lectures were the next most desired tools identified by respondents. Online modules can provide standardized patients, who can be optimized to provide a variety of traits and responses that practicing gynecologists may encounter in their practice. Online modules are a reasonable way to address both the unique healthcare problems of transgender patients as well as pronoun awareness and psychosocial issues associated with being transgender. These contextual concepts may be more difficult to grasp in a text-only learning environment such as a didactic presentation. By compiling information from a variety of resources (such as the WPATH, the University of California, San Francisco, Transgender Center for Excellence), an online module can bring together a manageable learning experience for the average Ob-Gyn residents without requiring them to seek out different sources, many of which are still only known to few programs. Only 45% of respondents knew of any organizations working with the transgender population, the majority of them only knew of the Transgender Center for Excellence.

Many in the medical field are aware that standardized patients invariably do not represent all patients in their target demographic scenario, but most recognize the value of standardized patients in providing exposure to common traits.^6^ The use of standardized patients allows residents to err without the risk of harming a patient. This is particularly relevant for transgender patients, who make up a small yet vulnerable population, with high levels of discrimination and mental health complications as a by-product.^3^ Insensitivity toward this community includes outright and perceived discrimination, lack of identification boxes appropriate for their sex/gender discrepancy, inappropriate pronoun usage, and lack of knowledge on the part of physicians and their healthcare affiliates. Educating residents in a safe space, such as in a standardized patient interaction, would be beneficial and a good supplement to any patient interaction they may receive in residency. When in-person standardized patient encounters are not available, an online learning module incorporating standardized patients would be a useful tool.

This survey does have its limitations. While all known program directors were invited to participate, it is possible that some were missed due to changing roles and contact information. In addition, while the response rate was 39.3%, a good response for an uncompensated survey, it was not possible to compare the respondents and nonrespondents to elucidate group differences due to the anonymous nature of the survey. Responder bias is possible as those who were familiar with, and more interested in, the CREOG objectives may have been more inclined to participate in the survey, thus leading to a higher percentage of respondents stating that they were familiar with the objectives. All program data were reported by program directors, who may or may not be aware of education regarding transgender health that is happening through unofficial faculty–resident interactions. Thus, some survey items may have been underestimated, reflecting residency programs where transgender education happens but is not a formal part of the curriculum. The survey also did not assess for the presence of institutional limitations on training residents to care for transgender patients due to such issues as institutional social ideologies. This may be a barrier to assess in future surveys.

## Conclusion

In the Washington Transgender Needs Assessment Survey, 32% of respondents listed insensitivity/hostility to transgender persons as a barrier to accessing care, as well as a fear of having their transgender status revealed.^7^ The transgender health CREOG objectives were created to ensure that graduating Ob-Gyn residents are equipped with the medical, communication, and cultural skills to be able to provide services to the transgender population. To fully achieve these goals, objective awareness must increase. While our hypothesis that program directors were unaware of these objectives was disproved, it does not discount the obvious need for improved educational tools for their programs. Providing these high-quality educational tools concurrently fills an identified need and ensures access to consistent educational concepts.

## Abbreviations Used

CREOG: Council on Resident Education in Obstetrics and Gynecology
Ob-Gyn: Obstetrics and Gynecology
WPATH: World Professional Association for Transgender Health
